# Access to healthcare for people experiencing homelessness in the UK and Ireland: a scoping review

**DOI:** 10.1186/s12913-022-08265-y

**Published:** 2022-07-13

**Authors:** Sarah McNeill, Diarmuid O’Donovan, Nigel Hart

**Affiliations:** Centre for Public Health, Queen’s University Belfast, Institute of Clinical Science, Block A, Royal Victoria Hospital, Belfast, Northern Ireland BT12 6BA

**Keywords:** Homelessness, Healthcare access, United Kingdom, Ireland

## Abstract

**Background:**

People experiencing homelessness (PEH) have poorer physical and mental health than the general population. They are also more likely to have less access to healthcare. These processes of access can be better understood using Levesque’s access framework which addresses both supply (service provision) and demand (user abilities).

**Methods:**

Following the Joanna Briggs Institute (JBI) guidelines, electronic peer-reviewed databases were searched in February 2022 for studies published since 2000 related to access to healthcare for PEH ages 16 and older in the United Kingdom (UK) and Ireland. Retrieved articles were screened and those eligible were selected for data extraction. Qualitative and quantitative studies were included.

**Results:**

Fifty-six papers out of 538 identified were selected and aliased. Six main themes were identified: staff education, flexibility of systems, service coordination, patient preparedness, complex health needs and holistic care. These relate to the Levesque access framework.

**Conclusions:**

Improving access to healthcare for PEH requires changes to how services are provided and how service-user abilities are supported.

**Supplementary Information:**

The online version contains supplementary material available at 10.1186/s12913-022-08265-y.

## Background

People experiencing homelessness (PEH) have poorer health than the general population; mortality rates are higher and morbidity trends show that infections, cardiovascular and respiratory conditions, premature ageing, high frailty scores are all more prevalent among homeless populations [1, 2]. Homeless populations are also disproportionately affected by mental health issues; with higher self-harm rates among PEH than housed counterparts [3]. Rates of hospital readmission are also higher for PEH [4].

Despite this, Julian Tudor Hart’s ‘Inverse Care Law’ holds for PEH; the availability of medical care varies inversely with the need for it in the population served [5, 6]. Emergency departments are frequently used by PEH but use of primary and preventative health services is relatively low, which may indicate that health is often not a priority until a crisis point is reached [7, 8].

Access to healthcare for PEH has been highlighted as a priority for research, both in the United Kingdom (UK) and Ireland [9, 10]. Prior to this scoping review, a preliminary search was conducted to ensure that there were no reviews published or in progress on the topic of access to healthcare for PEH in the UK and Ireland. The databases searched were Medline, Joanna Briggs Institute (JBI) Evidence Synthesis and the Cochrane Database. Therefore, this scoping review was conducted to provide a broad insight into the evidence available concerning access to healthcare for PEH, specifically within the UK and Ireland.

The theoretical understanding for access is taken from Levesque’s access framework [11], where access is described as a two-sided relationship with service provisions and service user abilities both affecting the process of access to healthcare. This framework is used as a template for discussion of results.

### Objectives

The objective of this scoping review is to assess the evidence from the published literature addressing access to healthcare for people experiencing homelessness in the UK and Ireland. Conclusions will highlight the main findings from all research in the review in order to make recommendations to improve access. The main question was:

‘What does the literature tell us about factors influencing access to healthcare for people experiencing homelessness in the United Kingdom and Ireland?’

## Methods

A protocol was designed before starting this scoping review, according to JBI recommendations [12]. This included background, eligibility criteria and methods for search strategy, evidence selection, data extraction, analysis and presentation. It was not published or registered.

### Eligibility criteria

**Fig. 1:**
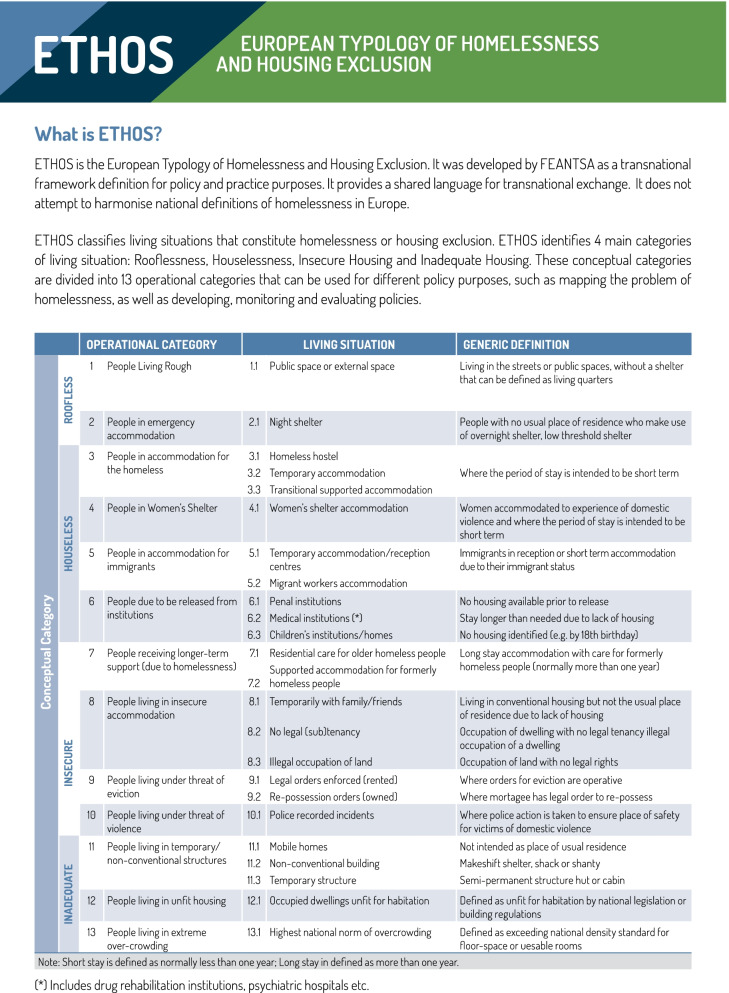
FEANTSA ETHOS Typology of Homelessness [13]

Eligibility criteria were decided using the Population, Concept, Context framework [12]. The population parameters were defined using the FEANTSA (European Federation of National Organisations working with the Homeless (French: Fédération Européenne d’Associations Nationales Travaillant avec les Sans-Abri)) ETHOS (European Typology of Homelessness and Housing Exclusion) groupings. This typology was first developed by FEANTSA in 2005 and re-designed in 2017 to use as a common ‘language’ allowing measurement and understanding of types of homelessness across Europe. It has four conceptual categories that are split into 13 organisational categories. For this review, the population includes adults (16 and older) experiencing homelessness using the definitions from the first 4 sub-categories of the FEANTSA framework, that is, two roofless sub-categories and the first two sub-categories of houseless (as indicated in Fig. 1) [13]. This includes people who are sleeping on streets or staying in short term accommodation.

The concept is access to healthcare, including primary and secondary healthcare as well as specific services. Levesque’s access framework was used for theoretical understanding: this, describes a two-way relationship in which service-user and provider both play a part [11]. Therefore, any study that includes aspects from Levesque’s framework from either service provider or service user side is included.

The context is the geographical nature of the limitations for this review, limited to UK and Ireland. The review was confined to these geographic limits as the results of this review will guide research in Northern Ireland, a region of the UK which has significant links to Ireland. A location specific scoping review adds value to this body of evidence as it allows researchers to understand the impact of culture in the selected countries. The results of this scoping review may contribute to further research design in this wider context.

Qualitative and quantitative research are included. Methodology is not a limiting factor, and all methods are included. Papers were excluded if published before 2000. This date was chosen to allow a wide range of data while ensuring included papers were relevant to health services today. An initial search showed most papers would be included in this date range. Opinion and commentary papers were not included.

### Search strategy

The literature search was finalised in February 2022. Following the JBI manual guidelines, key terms were entered into two databases and results were then scanned for further key terms to include in final search. The key terms were finalised in discussion with a subject librarian after several initial searches. Key terms were then entered into four databases: Medline, CINAHL, Web of Science and EMBASE. The key terms used were homelessness, healthcare access and United Kingdom (UK) or Ireland (Table 1). The exact synonyms for each of these were slightly different for each database as some databases have relevant subject headings and key phrases.

**Table 1:** Medline Search Terms

1	Homeless Persons/ or homeless.mp. or Homeless Youth/	13,089
2	homelessness.mp.	6053
3	houseless.mp.	12
4	roofless.mp.	18
5	Health Services Accessibility/ or health access.mp.	82,890
6	access to health service*.mp.	2645
7	health services access*.mp.	82,901
8	United Kingdom/	240,693
9	England/	91,969
10	Scotland/	25,700
11	Wales/	14,822
12	Northern Ireland/ or Ireland/	24,290
13	1 or 2 or 3 or 4	14,866
14	5 or 6 or 7	84,989
15	8 or 9 or 10 or 11 or 12	380,332
16	13 and 14 and 15	81

All papers from these searches were saved into EndNote and duplicates removed [14]. The final list was then transferred to Rayyan to facilitate the screening of titles and abstracts during the selection process [15]. Two reviewers did a blind independent screen of all papers using the predetermined eligibility criteria to make decisions. Conflicts were resolved through discussion with an additional reviewer.

### Data extraction

Data extraction and charting was completed by one reviewer using headings from the JBI manual. These headings were used for initial data charting with three papers as a sample and then edited slightly to suit the objectives of this scoping review. A data charting table was produced for approval by two additional reviewers. The full text of 88 papers was data-charted and 32 papers were excluded during this process, with any disagreement resolved through discussion by reviewers. After data-charting, results were coded. This started with margin notes that were then grouped together to form initial codes. This process was iterative, with codes developing as more papers were included. The codes were then grouped into themes, with agreement from two reviewers.

## Results

**Fig. 2:**
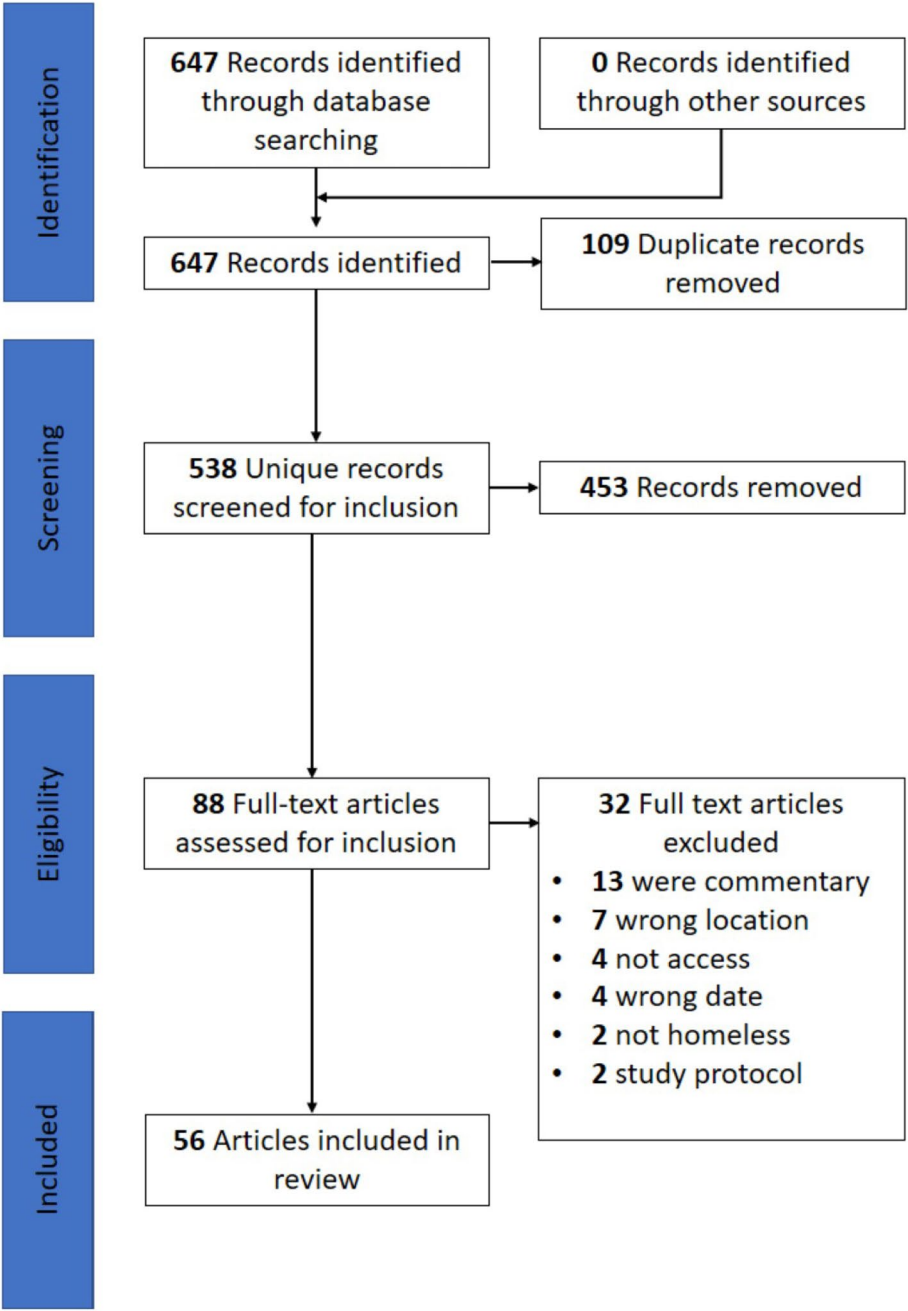
PRISMA flow diagram

Four databases produced 647 results in total (Fig. 2). There were 81 from Medline, 303 from CINAHL, 220 from Web of Science and 43 from Embase. These results were all downloaded to Endnote and duplicates removed, leaving 538 individual papers. When these papers were transferred to Rayyan, 453 were excluded based on title and abstract screening using eligibility criteria. Then, the full text of 88 papers were read and 32 more excluded. This resulted in 56 papers to be included in this scoping review (Additional file 1: Appendix 1).

The 56 selected papers included four that did not meet the agreed inclusion criteria but were included following researcher discussion as they were service evaluations that would add to the evidence as they offer insight into access to dental and pharmaceutical healthcare for PEH.

### Characteristics of evidence

The final results included 36 articles from England, 7 from Ireland, 7 from Scotland, 1 from Northern Ireland and 3 reporting on the wider UK. The final two were literature reviews that did not specify geographical location. Although this is not fully representative, it gives an overview of the general situation across the UK. Most studies took place in cities which limits the transferability of the results to rural areas. This is most noticeable for Ireland, where the only research included is from Dublin.

The participants were mostly PEH with 7 papers including health professionals. The sex of the PEH was reported in 20 studies. Fifteen had more male participants than female, 4 had more female and one had equal numbers of both.

The most common data capture method was semi-structured interviews with 22 papers reporting on results from interviews. Focus groups were used in 5 studies and questionnaires in 3. Other data collection methods used included freedom of information requests, surveys, census and observation. The health focus of the studies was varied and broad. The most common foci were dental hygiene, nursing intervention and end of life care.

The conclusions from papers were mostly similar, providing corroboration of themes. One notable difference was that two sources suggested that main services should be altered or improved rather than creating and using specialised services for PEH [16, 17]. One of these noted the issue that dedicated homeless services may not provide patients with specialist health services [16], while the other study concluded that reintegration into mainstream services created more challenges [17].

### Themes

Six themes were identified, namely staff education, flexibility of systems, service coordination, patient preparedness, complex health needs and holistic, patient centred care. These themes explore factors that influence access for PEH in the UK and Ireland. The themes overlap with aspects from Levesque’s access framework as outlined in the tables below (Tables 2 and 3).

**Table 2:** Papers categorised by themes and service provisions

	Approachability	Acceptability	Availability and Accommodation	Affordability	Appropriateness
Staff Education	[18] [19] [20] [21] [22] [23] [24] [25] [26] [27] [28] [29]	[30] [31] [32] [33] [34] [35] [36] [37] [38] [28] [29] [39]			[30] [18] [19] [31] [20] [32] [40] [21] [33] [23] [34] [35] [24] [36] [37] [25] [26] [27] [38] [28] [29] [41] [42] [39] [43]
Flexibility of Systems	[33] [42] [44]		[30] [20] [45] [46] [47] [48] [49] [40] [50] [21] [51] [52] [53] [33] [23] [8] [54] [34] [55] [16] [56] [35] [57] [24] [58] [38] [28] [42] [43] [59]		
Service Co-ordination					[18] [20] [45] [46] [32] [54] [16] [35] [24] [60] [25] [26] [27] [38] [28] [61] [29] [17] [44] [41] [39] [43] [59]
Patient Preparedness					
Complex Health Needs			[8] [56]		[24]
Holistic, Patient Centred Care				[31] [20]

**Table 3:** Papers categorised by themes and service user abilities

	Ability to Perceive	Ability to Seek	Ability to Reach	Ability to Pay	Ability to Engage
Staff Education					
Flexibility					
Service Co-ordination					
Patient Preparedness	[30] [31] [49] [40] [50] [51] [62] [38] [28]	[30] [31] [40] [50] [38] [43]	[20] [45] [54] [16] [56] [58] [29] [41]		[40] [34] [35] [58] [63] [43] [64] [59]
Complex Health Needs	[8] [56] [63] [28]	[65] [66]			[31]
Holistic, Patient Centred Care					

### Staff education

The attitudes and knowledge of staff and their impact on access were mentioned in 29 papers included in this review. Education was noted in most of these papers as a tool to improve both knowledge and attitudes of staff in both healthcare and hostel settings.

Education for health professionals was highlighted by both health professionals and PEH. Two papers used Freedom of Information requests to conclude that education on homelessness in English Healthcare Trusts was minimal if present at all [19, 23]. One qualitative study recommended that medical students should receive homeless specific education during their training [40]. PEH said that staff needed to be educated and become aware of the complexities they faced and to offer ‘realistic advice’ and ‘simple explanations’ [28–30].

Education for hostel staff was also requested by PEH, with aims to improve understanding of health needs and knowing when to escalate and refer patients to appropriate health services [21, 26, 27, 32]. It was acknowledged that this can be challenging as PEH tend to have complex health needs and their disease trajectory is often difficult to predict [18].

Several authors concluded that both healthcare and hostel staff should have a better understanding of navigating the health systems available for PEH, allowing them to signpost and direct PEH to appropriate services [20, 21, 24, 43].

One paper presented data in a narrative form, with the aim of educating the reader, allowing them to gain an empathetic understanding of the experiences of PEH and their struggle in accessing healthcare. The author used this technique to educate the reader and emphasise their key point that PEH are not ‘hard to reach’ but in fact services are ‘hard to reach’ for PEH. This was the only example where the author explicitly aimed to educate and challenge readers’ attitudes and perceptions [36].

### Flexibility of systems

The need for systems to be flexible and accommodating for PEH is the most common theme throughout, mentioned in 33 of the papers. Several authors specifically mentioned flexibility as a facilitator for access to healthcare for PEH, while others discussed the negative consequences of rigid systems. Key properties included location of services, appointments, and general practice (GP) registration.

The physical location of services was mentioned most often, featuring in 18 papers. In some cases, the services were mobile or outreach groups bringing the service to PEH rather than expecting them seek it out. In one case this was done using a GP-led bus that drove to areas where PEH would be and then invited them for health consultations [42]. Outreach and mobile approaches resulted in higher uptake or screening, vaccinations, and other health interventions [33, 44]. Location of services in relation to each other was also discussed, highlighting that access to all services may be easier if they are ‘all under one roof’ [16, 67]. However, authors acknowledged that having all services under one roof might lead to PEH missing out on specialist care that would be available if regular referral routes were used [16].

Rigid, individual appointment times were criticised in the results of several articles, suggesting instead that periods of time be set aside for homeless services. This would provide PEH, hostel staff or support workers with a window of opportunity to arrive at the health centre rather than a specific time slot. The flexibility around appointments also included giving out longer appointment times as many PEH have complex health needs that require longer than a usual GP time slot [21, 49].

Another noted flexibility issue was GP registration. A prospective patient may require identification and proof of address to register with a GP, which is often not possible for PEH [29, 67].

### Service co-ordination

Service co-ordination was highlighted as a factor that influences access to health services. It was mentioned in 24 articles included in this review. This included poor discharge planning, fragmentation of services, poor communication, lack of understanding of who is responsible for the health of PEH, difficult referral processes, and poor links between health systems and hostels or shelters [16, 20, 27, 28, 46]. A small number of authors noted the positive impact of good signposting. This refers to staff having an awareness of other available and relevant services and can direct PEH in the direction of support that will be helpful for them [38].

Discharge processes and transitions between services were reported to have a considerable impact on access for PEH. Poor communication or difficult discharge paperwork at transition stages was linked to patients falling through gaps in the system and losing access to healthcare [16, 29]. Some authors suggested a solution may be continuation of care planning and sharing notes between services [25, 41].

Provider accountability for PEH is lacking. They are often passed between services with no-one able to take on responsibility for all areas of their care [18]. There may also be PEH who leave hospital before receiving care, with no-one responsible for ensuring they follow through with recommended treatments [32]. Some papers highlighted the positive impact of a specialised role of district/public health nurse to fill this position. However, positive outcomes were noted to depend upon the individual who carried out the role [39].

Relationship and links between staff from different services was a facilitating factor for access to healthcare. This referred to both formal and informal relationships between hostel or shelter staff and healthcare providers as well as wider networks [27].

### Patient preparedness

Authors discussed the preparedness of patients for accessing healthcare. This included the awareness of need and desire to access healthcare, as well as the knowledge and ability to do so.

Negative experience of healthcare services was a barrier mentioned in most of the papers included in this review. This led to attachments to certain services and fear of relocation [40, 41]. Negative experiences included both social and physical setbacks, such as bad interactions with healthcare staff or side effects from medication [31]. One article suggested that providing an informal, flexible setting with non-judgemental staff and confidentiality would help to remove barriers that PEH had due to past negative experiences [38].

The ability to perceive health need is often lacking among this vulnerable group. Negative experiences may impact health beliefs and expectations and therefore the patient’s ability to perceive health need. In some cases, PEH were in denial that they had any health needs. This was from the perspective of PEH who were reflecting on health perceptions of homeless populations, including themselve s[30]. While other results stated that seeking healthcare is often not be a priority for PEH, from the perspective of PEH looking back on health experiences [51]. This was mainly when the health concern was not at crisis point, resulting in low attendance from PEH at preventative and primary health services [28].

While PEH may not always perceive or prioritise their own health needs, healthcare staff should still strive for patient autonomy. One article recommended asking patients what health support they want [34]. Improving autonomy can also include self-treatment of wounds and encouraging medication compliance [35, 63]. Another author emphasised the importance of empowerment and control over one’s health choices [43]. Empowerment and autonomy improve a patients ability to engage, however, perception of health need and desire for care can impact how much autonomy patients are given [11].

Knowledge of healthcare systems and services were identified as barriers to access for PEH [20, 29, 43]. This included knowledge of mainstream services as well as specialist services [16, 41]. There was also a lack of knowledge about preventative healthcare, such as health screening and health education [54].

Learning from peers about health behaviour and available services was acknowledged as a facilitator for improving access to healthcare [45, 49, 62]. Having a peer advocate or chaperone was recommended and supported by both staff and PEH. This model improved PEH attendance at appointments and helped with understanding during consultations [40, 58, 59, 64].

### Complex health needs

Several papers highlighted the issue of the complexities of health needs for PEH. This may be both a barrier to access or a result of poor access. Authors concluded that PEH have more complex needs than the general population and are more likely to experience comorbidities [8, 56]. Some authors focused on end-of-life care for PEH, reporting on the unpredictability of death and disease trajectory for PEH from the perspective of healthcare providers [18, 32] which may be exacerbated by low use of preventative health services and presenting with advanced illnesses [28, 63]. Some authors explored the complexity and vulnerability linked to high prevalence of mental instability and disjointed lifestyles [31]. Two papers found increased vulnerability when overlapped with other needs such as motherhood or learning disabilities [36, 58]. Other papers reported specific health concerns linked to homelessness, such as Hepatitis C, sexually transmitted illnesses, and alcohol use [55, 68, 69].

Illicit drug use was found as an added complexity with PEH, making accessing healthcare more difficult for PEH and creating issues for staff when treating them [65, 66]. Some PEH described a connection between their substance misuse and mental health needs but were required to be ‘clean’ from substance use before they could access mental health services, meaning they could not access services when they felt they needed it [67]. Illicit drug use also affects use of services after arrival, one hospital-based study concluded that withdrawal symptoms should be managed upon arrival to the Accident and Emergency (A&E) department to ensure access to healthcare was optimal [24]. Another author reported the issue of storing some drugs in hostels, sometimes causing PEH to miss out on medication that the general population could store safely [25]. Harm reduction healthcare was also included in conclusions, with authors discussing providing water for injection and needle exchange [65, 70].

### Holistic, person-Centred care

Several articles flagged the importance of addressing needs in a holistic, person-centred manner [33]. This included providing practical support alongside healthcare to fulfil basic needs such as hunger and shelter and to assist or encourage accessing other social or healthcare services [59, 61].

Person-centred care (PCC) is an evolving concept with frameworks that allow better understanding of the outcomes and aspects within PCC [71, 72]. The results of this scoping review included papers that discussed the need for human connection, relationship, trust, and the need for PEH to feel listened to [24, 34, 58]. These are all aspects of the therapeutic relationship highlighted in PCC frameworks [73].

Cost of health products was discussed in two papers, one in relation to contraception and one in relation to dental health products. The conclusions from these papers recommended making these products free to PEH [31, 62]. These papers were the only two to broach affordability, suggesting that affordability is not a usual barrier to access for PEH in the UK and Ireland.

## Discussion

### Levesque access framework

**Fig. 3:**
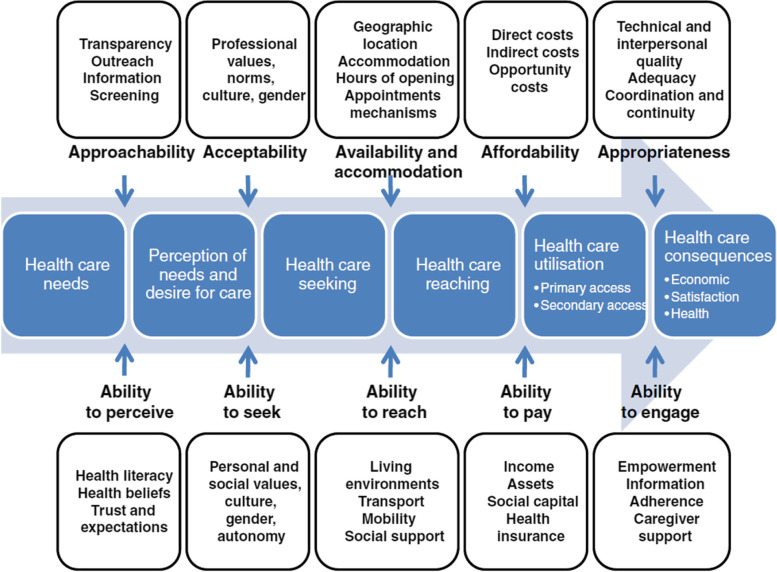
Levesque’s Access to Healthcare Framework

It is notable that these themes overlap with and relate to aspects of Levesque’s access framework (Fig. 3).

Directly copied [11]. Copyright VC 2013 by Levesque et al.; license BioMed Central Ltd.

Three of the themes (Staff Education, Flexibility and Co-ordination) link with the service accommodations from Levesque’s framework, one theme (Patient Preparedness) pertains to the abilities of the service-user and the final two themes (Complex Health Needs and Holistic, Person Centred Care) are more exclusive to access for PEH specifically but can be explored alongside Levesque’s framework [11].

Addressing staff education, knowledge and attitudes impacts several domains of the Levesque access framework, suggesting it is a vital component when improving access to healthcare. Education could improve approachability, with staff better educated about how to communicate effectively with PEH and get important information across. Acceptability could also be addressed by improving attitudes and professional values. Interpersonal qualities can also be improved by education: these would contribute to the appropriateness aspect of access.

The flexibility theme links distinctly with availability and accommodation, showing that practical, structural factors impact hugely on access. The approachability aspect relates to this theme as well, where location of services turns into outreach, increasing the chances of patients being able to approach the services.

Service Co-ordination is encompassed within Levesque’s appropriateness aspect. Coordination and continuity of services increase appropriateness of service provisions and improve efficiency as patients can be directly referred to the service they require rather than starting the access process again.

Both knowledge of health systems and peer support impact on all patient abilities outlined in Levesque’s framework. These include the abilities to perceive, seek, reach, pay and engage. The ability to pay does not feature in the articles reviewed. This may be due to the geographical limitations where all papers are from the UK and Ireland where welfare systems and the National Health Service means this population do not need to pay for healthcare out of pocket.

The final two themes, complex health needs and holistic, patient centre care do not link with service provisions or service-users’ abilities to access but instead affect the entire process of access. The complexities of health needs can be acknowledged in the first box in Levesque’s access framework, ‘health care needs’, impacting each step along the way. While holistic, patient centred care may transform one journey of access into several to meet the needs of a patient who needs access to more than one service.

Although the conclusions from this review are limited to UK and Ireland, they can be relevant to other similar contexts. A systematic review exploring experiences and needs of health and social care for PEH published in 2020 reported similar themes of interpersonal and structural dimensions with regards to access with the majority of included studies from USA and Canada [74].

### Recommendations

The identified themes provide opportunities for recommendations to improve healthcare access for PEH. Service provision can be improved by educating healthcare and hostel staff. Learning about homelessness and developing skills in communicating effectively with this population should improve attitudes and the distribution of information. The education should also increase knowledge of what services are available and how to signpost appropriately. Services could also introduce structural change, such as providing flexible appointment services in appropriate locations. Other structural change could include co-ordination between services with a focus on improving discharge planning or assigning designated staff members to be responsible for PEH, reducing the likelihood of patients falling through the cracks in services. Patient abilities must also be considered to improve access to healthcare for this population. The results suggest that patient’s abilities are improved with the addition of peer support. This may be through informal stories from peers about positive experiences or having a formally assigned peer to attend appointments with.

## Limitations

The geographical transferability of the results of this scoping review is limited. The majority of included studies are England based almost all are based in cities. Solutions for improving access to healthcare for PEH may differ across the UK and Ireland and strategies that work for big cities may not work for homeless populations in smaller urban or rural areas. The studies focus mostly on men experiencing homelessness and there is a relatively small number of health professionals’ voices included. Studies focusing on women and other stakeholders in other parts of the UK and Ireland would add greatly to the research body. This review also excludes the second two sub-categories of houseless in the European Typology of Homelessness and housing exclusion (ETHOS) typology (Fig. 1) i.e. migrant accommodation and people due to be released from institutions. Accessing health services for these groups presents a different set of challenges and will need to be explored separately [75, 76].

## Conclusion

Improving staff education, service flexibility and service co-ordination could improve service provision while supporting PEH could improve their service user abilities. The themes relate to Levesque’s access framework with the notable absence of affordability and ability to pay, potentially due to the geographical limits on the review. The final two themes must be included alongside Levesque’s framework when considering this vulnerable population. PEH have complex health needs, which creates a deeper need for access to services but also can hinder the entire process. PEH must also receive holistic care, meaning that PEH may be on several access journeys at the same time, trying to access healthcare, social care, or housing.

## Supplementary Information

**Additional file 1.**

## Data Availability

Please contact author Miss Sarah McNeill (smcneill22@qub.ac.uk) to request any supporting data.

## Abbreviations

A&E: Accident and Emergency
ETHOS: European Typology of Homelessness and housing exclusion
FEANTSA: European Federation of National Organisations working with the Homeless (French: Fédération Européenne d’Associations Nationales Travaillant avec les Sans-Abri)
GP: General Practice
JBI: Joanna Briggs Institute
PCC: Person Centred Care
PEH: People Experiencing Homelessness
UK: United Kingdom
