# Supplementary material for: Access to healthcare for people experiencing homelessness in the UK and Ireland: a scoping review
Source: BMC Health Serv Res. 2022 Jul 13;22:910. doi: 10.1186/s12913-022-08265-y (PMC9281084; doi:10.1186/s12913-022-08265-y)
Supplement: Supplementary file 1 — Additional file 1. [file 12913_2022_8265_MOESM1_ESM.docx]

Appendix 1 – Data Charting Table

| Citation | Author/Year | Title | Country/Context | Participants | Concept (& methods) | Key Findings |
| --- | --- | --- | --- | --- | --- | --- |
| [1] | (Chaturvedi, 2016) | Accessing psychological therapies: Homeless young people's views on barriers and facilitators. | Homelessness Charity in the UK | 7 16-25 year olds who attended 2 counselling sessions with organisation.  5 female  2 male | Individual semi-structured interviews (in hostel one research one participant)  ‘What do you think might be some reasons that might stop some young people from going to counselling?’  ‘What do you think can be done to overcome this?’  Thematic analysis | Barriers  -resistance to opening up  -stigma  -past experiences of health-seeking  -denial about needing help  -lack of familiarity with therapy  Facilitators  -patience and consistency of offer  -simple explanations  -demystifying and normalising counselling  -Emphasis on choice  -Informal settings |
| [2] | (Traynor, 2019) | Are district nurses well placed to provide equitable end-of-life care to individuals who are homeless? | UK, Canada, USA, Sweden, Australia | various | **Literature Review**  -District nurses suitability for EOL care for PEH | Difficulty in disease trajectory  -education (staff don’t know what end of life looks like in liver disease/Hep C)  Gaps in existing systems  -education  -unsuitable systems  -no-one accountable for care (pt passed from service to service)  -harm reduction services  District nurses can deliver training and they are well placed to deliver EOL care for PEH. |
| [3] | (Lucas et al., 2018) | Arrangements for adult service users who are homeless in English mental health trusts. | English mental health trusts | Adult service users of English mental health trusts  49 (of 51) Trusts responded with information that could be used. | Freedom of information requests sent to trusts regarding partnerships, specific projects, education/training, referral pathways, and intervention models/ approaches informing work with homeless service users. | Under half had dedicated programmes/ resources (inc. outreach, staff in hostels etc).  Others had some limited provisions (ie links between agencies) or none at all.  Education/training specific to homelessness was minimal if present. |
| [4] | (Shah et al., 2019) | The attitudes of homeless women in London towards contraception. | 2 homeless shelters in central London | 14 previously street homeless and/or living in temporary accomodation  (18-55 yo) | Convenience sampling.  Semi-structured face-to-face interviews. | Vulnerable  - mental stability, prostitution, disjointed lifestyle  Individual factors  -choosing which contraceptive method  Health Professionals  -stigma and made to feel unwelcome in clinics |
| [5] | (Paisi et al., 2019a) | Barriers and enablers to accessing dental services for people experiencing homelessness: A systematic review. | UK | 28 papers included  16+ experiencing homelessness, HCP or policy makers | **Systematic Review**  -barriers and enablers for accessing dental health services for PEH | Lived experience of homelessness  -complexity/chaotic lifestyles, emotions, knowledge  The healthcare system  -accessibility, organisation (cost, no fixed abode/hostel address, fragmentation of services, flexibility in working hours), staff encounters  Bottom-up approach – giving PEH a say in the development of services |
| [6] | (Jagpal et al., 2019) | Clinical Pharmacy Intervention for Persons Experiencing Homelessness: Evaluation of Patient Perspectives in Service Design and Development. | England – aiming to design patient-centred pharmacy services | 9 homeless people  7 male, 2 female | Public involvement sessions - Focus group with those in emergency shelters and one-to-one for those sleeping rough  Topic Guide for Questions | Acquaintance with pharmacy – most did not speak to their community pharmacist  Perceived benefits of a specific pharmacy service  -access, understanding, integration, minimising misuse, screening/diagnosis  Outreach  Peer Support  -word of mouth, when PEH use a service and tell others  Follow-up |
| [7] | (Whiteford and Simpson, 2015) | A codex of care: Assessing the Liverpool Hospital Admission and Discharge Protocol for Homeless People. | Liverpool, England | 7 hospital based clinicans  11 H&SC practitioners | Purposive sampling  Semi-structured interviews | Prioritisation  -Responses included acknowledging how important discharge is from the beginning of admission.  Communication between services  Partnership working  Post discharge care |
| [8] | (Doughty et al., 2018) | The Crisis at Christmas Dental Service: a review of an annual volunteer-led dental service for homeless and vulnerably housed people in London | Crisis at Christmas Dental Service service evaluation in the UK.  Aims to assess impact of CCDS. | 2,454 patients over the last 6 years between 23-29 December every year. | Data regarding treatments given plus patient feedback questionnaire. (staff placed to help with understanding -Likert scale) | 75% of patients use CCDS as their only dental treatment in the year.  97% patient satisfaction  Volunteers enjoyed their time, were making a difference and felt they could bring concerns to shift leader |
| [9] | (Lowrie et al., 2019) | A descriptive study of a novel pharmacist led health outreach service for those experiencing homelessness | Pop-up pharmacy service in Glasgow City Centre  Service evaluation | 52 patients mostly male | Pharmacists pop-up set up in homeless support venues  Random sampling. | Demographics of group were the key findings.  Descriptive study |
| [10] | (Shulman et al., 2018) | End-of-life care for homeless people: A qualitative analysis exploring the challenges to access and provision of palliative care | London, UK | Single homeless people (n=28)  Formerly homeless people (n=10)  Health- and social-care providers (n=48)  Hostel staff (n=30)  Outreach staff (n=10). | Opportunistic sampling.  Semi-structured interviews and focus groups | Complex behaviours  -leaving without treatment, staying in familiar setting, inexperience of HPs, requirements  Gaps in existing systems  -responsibility, holistic care,  Uncertainty and complexity  -unpredictable death, avoiding discussion |
| [11] | (Mills et al., 2015) | Engaging the citizenship of the homeless-a qualitative study of specialist primary care providers | Initially Scotland, then the broader UK | 13 interviews conducted with HPs who work with PEH  10 female, 3 male  9 GPs, 3 Nurses, 1 Health Visitor | Semi-structured phone interviews | Barriers to engagement  -rigid appointment, complexity of system  Difficulties engaging with Professionals  -stigma, low self-worth  System approaches to facilitate engagement  -location  Relationship approaches to facilitate engagement  -trust, helping with things other than health  Practice, patient, community and citizenship  -word of mouth, peer experience |
| [12] | (Paisi et al., 2020a) | Evaluation of a community dental clinic providing care to people experiencing homelessness: A mixed methods approach | Service evaluation  Peninsula Dental Social Enterprise, Plymouth, UK | 22 interviews (nine PDSE staff members, 11 patients, one support worker, and one volunteer) | Purposive sampling through a gatekeeper.  Semi-structured face-to-face interviews, topic guides used. | Barriers include:  -Past experiences  -Fear/phobia  -Patient-preparedness – priority setting/transient nature  Enablers:  -non-judgemental  -Clear explanations  -involvement from hostel staff  -chaperone  -patient preparedness  -reminders  -long appointment  -student involvement  -flexibility  -funding |
| [13] | (Caton et al., 2016) | Evaluation of a community dental service for homeless and 'hard to reach' people | Service evaluation of Revive Dental Care in Manchester, UK | 33 participants (patients, HPs, community centre staff) | Semi-structured interviews. | Past experiences  Outreach  Facilitators:  -Language barrier  -Approachable staff  Behaviour change  Looking forward |
| [14] | (Jenkins and Parylo, 2011) | Evaluation of health services received by homeless families in Leicester | 11 Hostels in Leicester City, UK | 49 Families  90% respondents female | Evaluating health visitor role  Survey | GP registration easy (noticeably different to single PEH)  Most wouldn’t seek help until fairly/very unwell  Would like a flexible/ apt that day/ drop in service  Specific services named (midwife, sexual health, child counselling)  Attitudes of staff important (friendly, helpful, approachable)  A&E attendance lower than single PEH  Having time to explain selves  Hostel staff didn’t know about available services |
| [15] | (Coles and Freeman, 2016) | Exploring the oral health experiences of homeless people: a deconstruction-reconstruction formulation | 4 Scottish Cities, Scotland, UK | 34 (self-identified homeless)  16-70yo  21 male, 13 female | 60 min interviews  Grounded Theory approach | Oral health deterioration goes hand in hand with general deterioration- rough sleeping= nowhere to brush teeth, for some, drugs are priority  Oscillation (intention to treat but missing appointments or other things (jail, drugs, other health service)  Reconstruction combines beginning an oral health routine, choosing a dentist |
| [16] | (Elwell-Sutton et al., 2017) | Factors associated with access to care and healthcare utilization in the homeless population of England | 19 areas of England | 2505 Non-statutory homeless (ie non-priority)  69% male  31% female | Quantitative  Used GP registration/refusal of GP and dental registration to determine primary care access | Lower rates of GP registration  Recent GP refusal led to A&E attendance but lower rates of hospital admission  Rough sleepers are less likely to be GP registered.  Hidden homeless higher GP registered |
| [17] | (Thomas and Barrett, 2021) | Fighting TB in underserved populations: homeless communities | Dorset, England, UK | 140 people came to health screening event | Health Screening Event  138 received CXR  86 TB test  78 bloodborne virus test | 5 with active TB (treated)  12 with latent TB (7 treated)  1 lung cancer  3 Hep C  High turn-out of PEH shows interest in personal health when barriers are removed. |
| [18] | (Neale and Kennedy, 2002) | Good practice towards homeless drug users: research evidence from Scotland | Scotland, UK | 12 interviews with staff (8f,4m) and 36 with clients (18f,18m)  6 case study agencies (3 drug services and 3 homelessness services) | Semi-structured interviews | Staff responses  Staffing (approachable), service delivery (pt centred, informal), support (outreach, aftercare and practical help), agency aims, agency environment  Client responses  -support (drugs, housing), agency environment (clean, friendly), staffing, agency aims (gain skills), service delivery |
| [19] | (Khan et al., 2019) | The GP role in improving outcomes for homeless inpatients | South London  Pathway team |  | **Literature Review** and data collected by Pathway team (Interviews by Pathway nurse)  Plus 10 structured interviews of pathway homeless team staff. | GP is viewed as pivotal in the team’s overall success.  Training – need for competency framework and specific training  Hospital staff liked having GPs involved in homeless care. |
| [20] | (Read, 2008) | Health and homelessness -- a whole-systems perspective | English Mental Health trusts, UK | 49 trusts provided information | Responses to Freedom of Information requests were analysed.  Asking for information concerning partnerships with external agencies, any projects, training, specific staff, referral pathways or intervention models that informed work with homeless service users | Just under half of the trusts had dedicated arrangements/resources – ie outreach teams, clinical staff located in homeless accommodation.  The rest either had limited arrangements or none at all.  Information on training/ improving staff awareness and knowledge was minimal if provided at all. |
| [21] | (Keogh et al., 2015) | Health and use of health services of people who are homeless and at risk of homelessness who receive free primary health care in Dublin | Dublin  Safetynet clinics – providing free healthcare to homeless people regardless of GMS status. Based in homeless shelters | 105 participants  75% male  52% had children | Comparison of data from people using SafetyNet in Dublin now vs data from 1997/2005.  133-item questionnaire to determine physical/mental health and use of health services (took about 45 mins to complete with researcher asking questions and writing down the participants verbal answers) | Highlights increased health needs in homeless population compared to general population. States that Dublin homeless population has similar health status as other homeless populations.  Increased use of GP services (average of 6.5 times in the last 6 months).  Increased diagnosis of health problems.  Increased prescription rates.  Almost a quarter said they would have gone to A&E if safetynet had not been available.  High levels of risky health behaviours (smoking etc) and high rates of suicidal thoughts and attempts. |
| [22] | (O'Carroll and O'Reilly, 2008) | Health of the homeless in Dublin: has anything changed in the context of Ireland's economic boom | Dublin. | 356 interviewees | Census of homeless adults, done in 2005. | 29% female in 1997, 39% female in 2005.  For those living homeless for more than a year, 5% female in 1997 and 58% female in 2005.  Increase in drug users becoming homeless causes an increase in targeted healthcare for PEH.  Mostly using secondary care. |
| [23] | (Swabri et al., 2019) | Health status of the homeless in Dublin: does the mobile health clinic improve access to primary healthcare for its users? | Dublin  Mobile Health Clinic – part of safetynet | 42 participants  90% male | Interviewer administered questionnaire to address characteristics and physical/mental health. | Mobile Health Clinic promotes access to primary health service (focuses on street homeless).  All demographics are noted to be the same as similar studies.  Recommendations include;  Screening and health education, focusing on disease prevention.  Build networks.  Should locate at weekends near an A&E. |
| [24] | (Ungpakorn and Rae, 2020) | Health-related street outreach: Exploring the perceptions of homeless people with experience of sleeping rough | London  Drop-in centres. | 10 participants  1 female | Semi-structured interviews | Health-related Street outreach offers human connection that reduces isolation and exclusion.  Street outreach as a bridge to help overcome barriers.  Right approach is essential – ie timing, location, verbal/non-verbal cues.  [DO: joint work with soup kitchens etc, first name badge with healthcare logo, come down to street level, respect personal space, open body language, ask people what they need and let them decide if they want it  DON’T: Work with enforcement, wear a uniform, wake people, demand personal information  and push healthcare agenda] |
| [25] | (Lambert et al., 2019) | 'HepCheck Dublin': An intensified hepatitis C screening programme in a homeless population demonstrates the need for alternative models of care | Dublin | 538 individuals were screened | Part of wider HepCare Europe initiative. | 37% testing positive for Hep C.(56% of those were new positives)  Active past 30 day drug use was common, unstable accommodation was most common barrier for accessing treatment.  Depression and anxiety, dental problems and respiratory conditions were most common reported health problems.  Hospital based appointments are inadequate for engaging this population. |
| [26] | (Dawes et al., 2017) | Homeless people's access to primary care physiotherapy services: an exploratory, mixed-method investigation using a follow-up qualitative extension to core quantitative research | London, UK  A homelessness specific GP surgery and a physio department receiving their referrals. | Healthcare records of 34 homeless patients.  5 staff interviewed from GP surgery (specifically for homeless). | Mixed methods design.  Physio referrals were matched with outcomes from physio dept. | Rate of attending initial appointment was lower than general population.  PEH lacked understanding about physiotherapy.  Traditional structures not suited to PEH.  Some GPs may have chosen to manage themselves rather than refer to a service that serves the whole community. |
| [27] | (McKenzie et al., 2019) | Homelessness—'It will crumble men': The views of staff and service users about facilitating the identification and support of people with an intellectual disability in homeless services | Scotland, UK | 16 staff members and 8 service users from homeless services | Semi-structured interviews to explore views on identification of support needs of homeless people with intellectual disability (ID) and the role of a screening questionnaire. | Some PEH did not know they had ID, or tried to cover it up.  Difficulty understanding.  Vulnerability.  Co-morbidities.  Environment and structure (forms, security number, call centres)  Limited time to listen and limited resources.  Building trusting relationships between services and users  Is it helpful to have a diagnosis if the healthcare worker isn’t going to support individual through it. |
| [28] | (Morton, 2017) | How working differently improved homeless people's access to healthcare | Staffordshire health inclusion team | 20 patients who were frequent A&E attenders (more than 3 a year) | Evaluation of inclusion health team – purpose to improve physical health of homeless people.  Each patient, the number of attendances in 12 months before being allocated to the health inclusion team and then 12 months after. | Flexibility  No appointments  Location (in hostel)  Lack of stigma  Transport provided  Individual, not the addiction  Joint appointments (methadone and wound dressing)  Attitudes of staff  Self treatment of dressings (found to improve compliance) |
| [29] | (Story et al., 2014) | Influenza vaccination, inverse care and homelessness: cross-sectional survey of eligibility and uptake during the 2011/12 season in London | London, UK  27 Homeless hostels, day centres and drug services.  Find and Treat service | 445 clients took part in the survey | Assess the homeless people visited by Find and Treat find eligibility for influenza vaccination. | 41.8% of those surveyed were eligible for influenza vaccination.  16-64 : 23.7% uptake rate compared with 53.2% nationally. (this group were nearly 3 times more likely to be eligible for vaccine)  65+ : uptake was 42.9% compared with 74% nationally. |
| [30] | (Schneller, 2012) | Intermediate care for homeless people: results of a pilot project | South London, UK  Lessons to be learned from a Nurse-led intermediate care pilot project – 120 bed homeless hostel |  | Evaluation of service that aimed to reduce A&E attendance, ambulance call outs and use of acute services. | During the pilot study at St Mungos, hospital admissions dropped by 77% and A&E attendances 52% and ambulance call outs 67%.  These same drops were not seen in other hospitals in the area.  Actions:  -Have frequent attendees documented  -Establish what type of homeless and contact hostel if applicable  -Reduce drug/alcohol withdrawal symptoms  -Recognise literacy issues  -Trust takes time  -Provide local homeless shelters with information  -Build links with GPs  -Refer clients  -Resource pack for new staff  -mental health follow up |
| [31] | (Barrow and Medcalf, 2019) | The introduction of a homeless healthcare team in hospital improves staff knowledge and attitudes towards homeless patients | Gloucestershire Royal Hospital, UK | Audit of homeless patients | Examining discharge outcomes of homeless in hospital.  10 question questionnaire to assess staff knowledge and attitudes (done twice, three years apart after new homeless patient policy) | Intervention was put in place to have a homeless inreach team to coordinate discharge planning (one housing officer and one part time nurse who escorted PEH to temporary accommodation and follow up)  Hospital is valuable point of contact as these people are usually hard to reach but they are overrepresented at hospital.  Effective coordination between hospital and homeless services saves money and improves patient care. |
| [32] | (Hebblethwaite et al., 2007) | Investigating the experiences of people who have been homeless and are in contact with learning disability services | England, UK | 14 participants | Semi-structured interviews at their current accommodation. Content analysis used. | Homeless people with learning disabilities are more vulnerable to exploitation from other residents.  Access to healthcare is facilitated by support in arranging and attending appointments.  4 felt they had received helpful advice  1 said someone had listened to him  4 had support in making appointments and 4 had been accompanied.  Registering with GP while in temporary accommodation = difficult. |
| [33] | (Fordham, 2015) | The lived experience of homeless women: insights gained as a specialist practitioner | Bedfordshire?  England | Women who attended clinics or lived in hostels. | Illuminate lived experiences of homeless women who attended clinics or lived in hostels.  Narrative study using reflective practice guidance. | Tells the story of ‘Mary’ a pregnant homeless woman through narrative story telling. Invokes emotion and disbelief in the treatment and service failures.  Most women included were mothers.  Stories intensify women’s struggles.  Aims to engage other health professionals to see PEH not as hard to reach but the health services are hard to reach. |
| [34] | (O'Carroll and Wainwright, 2019) | Making sense of street chaos: an ethnographic exploration of homeless people's health service utilization | Homeless services, Dublin, Ireland |  | Ethnographic observations and 47 semi-structured interviews, 2 focus groups. | PEH tend to present late in illness and default early from treatment.  Low use of primary, preventative, and outpatient  High use of emergency and inpatient  Poor compliance with medication  Avoidance of psychiatric services  External barriers:  Physical (distance)  Administrative  Attitudes  +Conversations of exclusion  Internal barriers:  Cognitive  Emotional |
| [35] | (Paisi et al., 2019b) | Management of plaque in people experiencing homelessness using 'peer education': a pilot study | Plymouth, UK | Baseline sample included 24 people.  Convenience sample from people living in residential centre, had to provide informed consent without their capacity being compromised by drugs or alcohol. | After initial clinical assessment, dentist demonstrated toothbrushing to each person.  A Groundswell peer educator provided basic oral health information.  Participants given toothbrush, paste, timer and leaflet. | None of the participants were registered with a dentist.  Tobacco and sugar intake was high and toothbrushing low.  Embarrassment and self-consciousness stopped affected oral health related quality of life and seeking treatment.  Peer researchers and educators are experts by experience and increases acceptability of project by participants. |
| [36] | (Hewett et al., 2011) | Morbidity trends in the population of a specialised homeless primary care service | Leicester | 131 patients  17-64 yo  15.8% female | Annual progress reports on developing services. | Average age of death is 40.5 yo.  Alcohol is cause of death for 47.3%.  Frequencies of mental health diagnoses are stable, marked drop in episodes of self-harm and suicide attempts – perhaps indicates improved access to psychiatric help through homeless mental health and crisis-resolution teams. |
| [37] | (Miller and Appleton, 2015) | Multiple exclusion homelessness: is simplicity the answer to this complexity? | Complex Needs Service, West Midlands, England, UK |  | Semi-structured interviews, analysis of referral and outcome data, focus groups with frontline staff and interviews with people living in the service. | Characteristics of service that were good:  -staff (relationships, flexibility, generous staffing numbers, willingness to respond, low turnover)  This service was to act as an integrator between individuals and services. |
| [38] | (Harris et al., 2020) | Navigating environmental constraints to injection preparation: the use of saliva and other alternatives to sterile water among unstably housed PWID in London | London, UK | Questionnaire and urinalysis (455)  Qualitative Interview (32) | Harm Reduction | Public Toilets closed and drug treatment services closed – people using alcohol, rain water and saliva for drug injecting.  Messaging for using safe water must be improved.  Increased supply of water for injection is also encouraged. |
| [39] | (McGregor et al., 2018) | Nurse-led sexual health clinics in hostels for homeless people | 3 St Mungos hostels in North London.  Clinics were located within each hostel once a week between 12.30 – 3pm. | 161 people.  Initially only targeted females but males asked if they could also attend and were later included. |  | 59 STIs diagnosed among the 161 clients.  No HIV positive.  5 positive Hep B and/or C  Most common contraceptive was condom.  4 received emergency contraception and 5 tested - one positive.  Themes found in interviews:  Risk to sexual health  Domestic Abuse/Violence  Services  stigma/embarrassment. Overall knowledge was lacking. Afraid of meeting new staff. Opportunities for communication. Did not attend unless significant problem. On site care provision seen as positive (more flexible). |
| [40] | (Webb, 2018) | Nursing management of people experiencing homelessness at the end of life | No geographical boundary |  | **Literature Review**  Homelessness and palliative care.  Suggests ways of working to improve access to palliative care. | CQC report acknowledged that PEH were not receiving good, personalised care for EoL (2016).  Barriers – prejudice of staff, mistrust of healthcare providers, inflexible policies, HP lack of awareness.  Drug Misuse- hostels can’t store controlled drugs (suggestion of lockable cabinet?), Homeless Link provides guidance, pharmacies may help, transdermal patches.  Collaborative working- hostel staff and healthcare, education for hostel staff in hostel, online learning, parallel planning (hostels are for recovery = EoL conversations are difficult).  Future- can’t be assumed that PEH EoL priorities are the same as general population, |
| [41] | (Melvin, 2004) | A nursing service for homeless people with mental health problems | Chester, UK |  | **Commentary** on a mental health clinical coordinator for homelessness (author is the practitioner).  A service that bridges the gap between primary and secondary. | Hostel Liaison- Helps identify clients, and deliver effective care, education and training for hostel staff was popular.  Prison Partnerships- allows collaborative care planning on release.  Assertive Outreach Approach allowed access to primary and secondary care. Ie going to clients. |
| [42] | (Taylor et al., 2007) | Organisational issues facing a voluntary sector mental health service for homeless young people | Whole UK |  | Service Evaluation- voluntary organisation for youth homeless (16-25) (Foyer Federation).  Examining experiences of inter-agency practitioners.  Strong Minded project – mental health coordinators (MHC)  19 semi-structured interviews with 5 MHCs, 10 foyer staff and 4 local MH professionals who worked with Strong Minded. | Differing priorities (foyers were not focused on mental health but SM was, some staff weren’t sure the ethos’s matched).  Referral process was difficult as hostel workers needed good relationships and needed to spot mental health concerns (they stated MH was difficult to understand)  NHS/voluntary sector collaboration.  Not high risk enough for services.  Isolated MHC – not part of a team. |
| [43] | (Johnsen et al., 2021) | Outreach-based clinical pharmacist prescribing input into the healthcare of people experiencing homelessness: a qualitative investigation | Glasgow, UK | 40 PEH (current or recent homeless 33m, 7f), 4 staff, 10 representatives of stakeholder agencies. | Semi-structured Interviews.  Transcripts analysed systematically via thematic or framework analysis.  Perceptions of an outreach service, delivered by prescribing pharmacists. | Service was effective at case finding and engaging with patients – friendly, informal, non-judgemental staff attitudes, time of appointment (listened to), convenience and immediate action, Signposting.  Helped pts overcome barriers – informal and flexible service, confidentiality, didn’t have to go to somewhere with a bad experience, impaired mobility.  Enabled immediate diagnosis and prescription of medication – understanding effect of medication,  Capitalised on opportunity to address pt healthcare needs. |
| [44] | (Rae and Rees, 2015) | The perceptions of homeless people regarding their healthcare needs and experiences of receiving health care | 2 local homeless services in ?England? | 2 women, 12 men | Semi-structured interviews using open ended questions  Colazzi’s eight step analysis framework. | Expressed health need- priority wasn’t health until crisis point, safety, lack of resources.  Healthcare experiences- difficult registering with GP (new), flexibility was good, negative experiences included unrealistic advice, poor discharge planning from hospital/ prison.  Attitudes- responsibility. |
| [45] | (Wyatt, 2017) | Positive outcomes for homeless patients in UCLH Pathway programme | Service evaluation of Pathways to see if A&E reduction, admission or bed days.  England | Audit 90 days before and after pathway care for 400 homeless patients. | 58% of patients were given housing and shelter advice or repatriation assistance.  Housing support officers.  Taxis, transport tickets given upon discharge.  Collaboration with charities and integrated discharge team.  2 beds in a local hostel as respite post discharge before transition back into community. | Pathway reduces A&E use, hospital admission and bed days. |
| [46] | (Gunner et al., 2019) | Provision and accessibility of primary healthcare services for people who are homeless: a qualitative study of patient perspectives in the UK | West Midlands, England, UK | 22 PEH  15m, 5f  14 white, 2 asian, 2 black, 1 mixed, 1 prefer not to say | Semi-structured interviews, transcribed and analysed using thematic framework approach.  Exploring perceptions of PEH on provision and accessibility of primary healthcare services. | Service Delivery:  -Difficulties registering at GP practices.  -Integration of services- signposting and specific mental health services.  -Continuity of Care- transitions across care services, sharing medical records  -Waiting times and appointment lengths  Patient-Related Factors:  -Pts knowledge and awareness of primary health services  -Pts skills and health literacy  -Pts resources  -Pts feelings and emotions  Social Exclusion and stigma  GP awareness of PEH complexities |
| [47] | (Anderson and Ytrehus, 2012) | Re-conceptualising Approaches to Meeting the Health Needs of Homeless People |  |  | Comparing Norway and Scotland | Issues of reintegrating into mainstream services after accessing specific services.  Are specific services problematic? |
| [48] | (Gray, 2007) | Referral patterns and access to dental services of people affected by homelessness in Dublin, Ireland | Dublin, Ireland | 237 charts  69% male  31% female | A review of database of records or patients who had attended the dental service for the homeless in the previous 12 months. | Notable differences between this study results and the results from the ‘Counted In’ Study conducted previously:  Female – 6% of female patients were over 40, compared to 33% of females over 40 in ‘Counted In’. – concluded that service needs to be more accessible for females over 40. However, is it that the original numbers were incorrect or the numbers are so transient that you can’t compare one to the next? Or are females less likely to access certain services?  Hostels with good communication links were more likely to refer patients.  Day centre referral was more influenced by outreach screening.  Self referrals = 16%  Concludes that targeting certain populations is necessary because homeless population isn’t homogenous. |
| [49] | (Smith et al., 2018) | Relocating patients from a specialist homeless healthcare centre to general practices: a multi-perspective study | Scotland | 17 Patients and 19 healthcare staff (nurses, GPs, substance misuse workers, admin, pharmacy staff) | Exploring barriers and facilitators or the process of transition between homeless services and main health service.  Semi-structured face-to-face and telephone interviews | Beliefs and consequences regarding relocation:  Continuation of healthcare needs, new staff/policies, ability to integrate  Patient Intention to relocate: reluctance, previous negative experiences  Environmental context and resources in relation to the care of patient/ assessing patient eligibility: pts housing status, communication between services, pts access to phone/WiFi, access to podiatry/dentistry  Patient skills: knowledge; of eligibility, mainstream system/structure/rules.  Social and professional role/identity of staff and patients: self-identifying as homeless, pts not perceiving homeless specialist as specialist, pharmacist as potential. Self-esteem/confidence of pts  Emotional attachment to SHHC: need family/staff etc to promote reintegration, pt attachment to certain services |
| [50] | (O'Carroll et al., 2017) | A review of a GP registrar-run mobile health clinic for homeless people | Dublin, Ireland  Mobile health clinic | 116 people who access the mobile health clinic filled out a questionnaire. (57% male, 43%female)  2 focus groups were conducted with 6 and 14 GP registrars who worked on the bus. | Questionnaire and focus groups to assess the mobile run health bus for PEH in Dublin. | 52% did not have a medical card (needed to access the free healthcare they are entitled to).  40% difficulty in accessing GP services.  Clients valued convenience and non-judgemental atmosphere.  GPs felt working on the bus:  Challenged their stereotypes of PEH and humanised.  Increased their knowledge of PEH issues.  Environment promoted positive relationship/attitudes.  Made staff think about improving their own practice to increase access. |
| [51] | (Poulton et al., 2006) | The role of the public health nurse in meeting the primary health care needs of single homeless people: a case study report | Northern Ireland case study of nursing roles using public health framework | 6 innovative roles were selected as representative across different nursing specialities | Semi-structured interviews and observation | The role fitted in a public health framework.  Role involved; targeting inequalities in health, addressing health needs, facilitating access, skills to meet needs, partnership working, health promotion, health protection and influencing policy and strategy development.  The success of post is attributed to the post holder (their personal attitudes etc)  Networking skills |
| [52] | (Paisi et al., 2020b) | Strategies to improve oral health behaviours and dental access for people experiencing homelessness: a qualitative study | Plymouth, England, UK  Oral/Dental Health | 11 british males experiencing homelessness and 12 other stakeholders from various professional backgrounds | Focus Groups with the people living in homeless centre and semi-structured interviews with other stakeholders.  Following an inductive approach and using thematic analysis. | Themes Identified:  Awareness and Empowerment for both PEH and staff   - Knowledge - Signposting - Targeting   Supportive Environment and Dental Health System   - Provision of free dental equipment (toothbrushes) - Funding - Accompanying to appointment   Flexible and Holistic Care  Outreach and Community Engagement  Collaboration with other health and social services  Effective Communication |
| [53] | (Fountain et al., 2003) | Unmet drug and alcohol service needs of homeless people in London: A complex issue | London, England, UK | 389 PEH  81% male and 19% female | Community survey and structured questionnaire  This study feels quite out of date- sample is mostly whit (does this mean its more like NI?), really focuses on drug/alcohol services/addictions and not other health needs | 54% had first become homeless at 18 or younger  Service uptake:  Drug/alcohol dependencies;  Needle exchange 85% uptake but next highest was methadone at 28%  Knowledge of drug services was relatively high (72%/78% for drop in specific heroin/advice&information centre). Numbers dropped for other services eg self help group/ day program  Reasons given for not using these services was that PEH did not want to stop drugs/alcohol |
| [54] | (MacLellan et al., 2017) | Using peer advocates to improve access to services among hard-to-reach populations with hepatitis C: a qualitative study of client and provider relationships | Study embedded with the HALT study – a hepatitis C RCT.  London, UK | 5 peer advocates | Narrative interviews. | Peer advocates build rapport with clients through disclosing personal details of their lives.  Rapport  Self-Disclosure  Advocate and HPs in the same group working toward same goal |
| [55] | (Cameron et al., 2009) | Working across boundaries to improve health outcomes: a case study of a housing support and outreach service for homeless people living with HIV | Housing Support Outreach and Referral  Considering the role of housing support in improving health.  Bristol, England, UK |  | Evaluation of programme. 15 months, 56 referrals and 27 accepted.  Plus interviews with professionals | Local joint working context- HIV/PCTs already worked together.  Voluntary Sector- well established involvement of statutory/non statutory  Support Worker- flexibility, bridging/signposting, accompanying people to apts,  Need for coordinated response from all sectors  Initial housing needs then dealing with health concerns |
| [56] | (Mc Conalogue et al., 2021) | Homeless people and health: a qualitative enquiry into their practices and perceptions | Convenience sample of homeless participants, recruited through non-health organisations.  Gloucestershire, England | 28 homeless people  54% in supported housing  39% rough sleeping  Of total, 29% female  Of those rough sleeping, 18% female | Semi-structured interviews  How homeless people understand, manage and prioritise health. | Understanding of health needs was present but people felt they had no control over factors that would support health  Difficulty accessing mainstream services  Looked down upon by staff  Base level needs must be met before health can be a priority  Trauma associated with Adverse Childhood Experiences must be understood. |

## References

1. Chaturvedi S. Accessing psychological therapies: Homeless young people's views on barriers and facilitators. Counselling & Psychotherapy Research. 2016;16(1):54-63.

2. Traynor R. Are district nurses well placed to provide equitable end-of-life care to individuals who are homeless? British Journal of Community Nursing. 2019;24(4):165-72.

3. Lucas S, Archard PJ, Tangen J, Murphy D. Arrangements for adult service users who are homeless in English mental health trusts. Mental Health Review Journal. 2018;23(1):64-71.

4. Shah P, Koch T, Singh S. The attitudes of homeless women in London towards contraception. Primary Health Care Research and Development. 2019;20.

5. Paisi M, Kay E, Plessas A, Burns L, Quinn C, Brennan N, et al. Barriers and enablers to accessing dental services for people experiencing homelessness: A systematic review. Community Dent Oral Epidemiol. 2019;47(2):103-11.

6. Jagpal P, Barnes N, Lowrie R, Banerjee A, Paudyal V. Clinical Pharmacy Intervention for Persons Experiencing Homelessness: Evaluation of Patient Perspectives in Service Design and Development. Pharmacy. 2019;7(4).

7. Whiteford M, Simpson G. A codex of care: Assessing the Liverpool Hospital Admission and Discharge Protocol for Homeless People. International Journal of Care Coordination. 2015;18(2-3):51-6.

8. Doughty J, Stagnell S, Shah N, Vasey A, Gillard C. The Crisis at Christmas Dental Service: a review of an annual volunteer-led dental service for homeless and vulnerably housed people in London. British Dental Journal. 2018;224(1):43-7.

9. Lowrie F, Gibson L, Towle I, Lowrie R. A descriptive study of a novel pharmacist led health outreach service for those experiencing homelessness. International Journal of Pharmacy Practice. 2019;27(4):355-61.

10. Shulman C, Hudson BF, Low J, Hewett N, Daley J, Kennedy P, et al. End-of-life care for homeless people: A qualitative analysis exploring the challenges to access and provision of palliative care. Palliative medicine. 2018;32(1):36-45.

11. Mills ED, Burton CD, Matheson C. Engaging the citizenship of the homeless-a qualitative study of specialist primary care providers. Family Practice. 2015;32(4):462-7.

12. Paisi M, Baines R, Worle C, Withers L, Witton R. Evaluation of a community dental clinic providing care to people experiencing homelessness: A mixed methods approach. Health Expectations. 2020;23(5):1289-99.

13. Caton S, Greenhalgh F, Goodacre L. Evaluation of a community dental service for homeless and 'hard to reach' people. British Dental Journal. 2016;220(2):67-70.

14. Jenkins M, Parylo C. Evaluation of health services received by homeless families in Leicester. Community Practitioner. 2011;84(1):21-4.

15. Coles E, Freeman R. Exploring the oral health experiences of homeless people: a deconstruction-reconstruction formulation. Community dentistry and oral epidemiology. 2016;44(1):53-63.

16. Elwell-Sutton T, Fok J, Albanese F, Mathie H, Holland R. Factors associated with access to care and healthcare utilization in the homeless population of England. Journal of Public Health. 2017;39(1):26-33.

17. Thomas D, Barrett S. Fighting TB in underserved populations: homeless communities. British Journal of Nursing. 2021;30(1):24-6.

18. Neale J, Kennedy C. Good practice towards homeless drug users: research evidence from Scotland. Health & social care in the community. 2002;10(3):196-205.

19. Khan Z, Haine P, Dorney-Smith S. The GP role in improving outcomes for homeless inpatients. Housing, Care & Support. 2019;22(1):15-26.

20. Read S. Health and homelessness -- a whole-systems perspective. Housing, Care & Support. 2008;11(1):7-10.

21. Keogh C, O'Brien KK, Hoban A, O'Carroll A, Fahey T. Health and use of health services of people who are homeless and at risk of homelessness who receive free primary health care in Dublin. BMC Health Services Research. 2015;15:58.

22. O'Carroll A, O'Reilly F. Health of the homeless in Dublin: has anything changed in the context of Ireland's economic boom. European Journal of Public Health. 2008;18(5):448-53.

23. Swabri J, Uzor C, Laird E, O'Carroll A. Health status of the homeless in Dublin: does the mobile health clinic improve access to primary healthcare for its users? Ir J Med Sci. 2019;188(2):545-54.

24. Ungpakorn R, Rae B. Health-related street outreach: Exploring the perceptions of homeless people with experience of sleeping rough. J Adv Nurs. 2020;76(1):253-63.

25. Lambert JS, Murtagh R, Menezes D, O'Carroll A, Murphy C, Cullen W, et al. 'HepCheck Dublin': An intensified hepatitis C screening programme in a homeless population demonstrates the need for alternative models of care. BMC Infectious Diseases. 2019;19(1):128.

26. Dawes J, Deaton S, Greenwood N. Homeless people's access to primary care physiotherapy services: an exploratory, mixed-method investigation using a follow-up qualitative extension to core quantitative research. Bmj Open. 2017;7(6).

27. McKenzie K, Murray G, Wilson H, Delahunty L. Homelessness—'It will crumble men': The views of staff and service users about facilitating the identification and support of people with an intellectual disability in homeless services. Health & Social Care in the Community. 2019;27(4):e514-e21.

28. Morton J. How working differently improved homeless people's access to healthcare. Primary Health Care. 2017;27(8):25-9.

29. Story A, Aldridge RW, Gray T, Burridge S, Hayward AC. Influenza vaccination, inverse care and homelessness: cross-sectional survey of eligibility and uptake during the 2011/12 season in London. Bmc Public Health. 2014;14.

30. Schneller K. Intermediate care for homeless people: results of a pilot project. Emergency Nurse. 2012;20(6):20-4.

31. Barrow V, Medcalf P. The introduction of a homeless healthcare team in hospital improves staff knowledge and attitudes towards homeless patients. Clinical Medicine. 2019;19(4):294-8.

32. Hebblethwaite A, Hames A, Donkin M, Colman M, Forsyth A. Investigating the experiences of people who have been homeless and are in contact with learning disability services. Learning Disability Review. 2007;12(3):25-34.

33. Fordham M. The lived experience of homeless women: insights gained as a specialist practitioner. Community Practitioner. 2015;88(4):32-7.

34. O'Carroll A, Wainwright D. Making sense of street chaos: an ethnographic exploration of homeless people's health service utilization. International Journal for Equity in Health. 2019;18(1):N.PAG-N.PAG.

35. Paisi M, Witton R, Burrows M, Allen Z, Plessas A, Withers L, et al. Management of plaque in people experiencing homelessness using 'peer education': a pilot study. British Dental Journal. 2019;226(11):860-6.

36. Hewett N, Hiley A, Gray J. Morbidity trends in the population of a specialised homeless primary care service. British Journal of General Practice. 2011;61(584):200-2.

37. Miller R, Appleton S. Multiple exclusion homelessness: is simplicity the answer to this complexity? Journal of Integrated Care. 2015;23(1):23-34.

38. Harris M, Scott J, Hope V, Wright T, McGowan C, Ciccarone D. Navigating environmental constraints to injection preparation: the use of saliva and other alternatives to sterile water among unstably housed PWID in London. Harm Reduction Journal. 2020;17(1).

39. McGregor F, Stretch R, Cannon E, Robinson A, Shawe J. Nurse-led sexual health clinics in hostels for homeless people. Nurs Times. 2018;114(5):42-6.

40. Webb WA. Nursing management of people experiencing homelessness at the end of life. Nurs Stand. 2018;32(27):53-63.

41. Melvin P. A nursing service for homeless people with mental health problems. Mental Health Practice. 2004;7(8):28-30.

42. Taylor H, Stuttaford M, Vostanis P. Organisational issues facing a voluntary sector mental health service for homeless young people. Journal of Integrated Care. 2007;15(1):37-47.

43. Johnsen S, Cuthill F, Blenkinsopp J. Outreach-based clinical pharmacist prescribing input into the healthcare of people experiencing homelessness: a qualitative investigation. Bmc Health Services Research. 2021;21(1).

44. Rae BE, Rees S. The perceptions of homeless people regarding their healthcare needs and experiences of receiving health care. J Adv Nurs. 2015;71(9):2096-107.

45. Wyatt L. Positive outcomes for homeless patients in UCLH Pathway programme. British Journal of Healthcare Management. 2017;23(8):367-71.

46. Gunner E, Chandan SK, Marwick S, Saunders K, Burwood S, Yahyouche A, et al. Provision and accessibility of primary healthcare services for people who are homeless: a qualitative study of patient perspectives in the UK. The British journal of general practice : the journal of the Royal College of General Practitioners. 2019;69(685):e526-e36.

47. Anderson I, Ytrehus S. Re-conceptualising Approaches to Meeting the Health Needs of Homeless People. Journal of Social Policy. 2012;41:551-68.

48. Gray R. Referral patterns and access to dental services of people affected by homelessness in Dublin, Ireland. Journal of Disability & Oral Health. 2007;8(2):51-6.

49. Smith KG, Paudyal V, MacLure K, Forbes-McKay K, Buchanan C, Wilson L, et al. Relocating patients from a specialist homeless healthcare centre to general practices: a multi-perspective study. British Journal of General Practice. 2018;68(667):e105-e13.

50. O'Carroll A, Irving N, O'Neill J, Flanagan E. A review of a GP registrar-run mobile health clinic for homeless people. Ir J Med Sci. 2017;186(3):541-6.

51. Poulton B, McKenna H, Keeney S, Hasson F, Sinclair M. The role of the public health nurse in meeting the primary health care needs of single homeless people: a case study report. Primary Health Care Research & Development (Sage Publications, Ltd). 2006;7(2):135-46.

52. Paisi M, Witton R, Withers L, Plessas A, Burrows M, Morrison S, et al. Strategies to improve oral health behaviours and dental access for people experiencing homelessness: a qualitative study. British Dental Journal. 2020.

53. Fountain J, Howes S, Strang J. Unmet drug and alcohol service needs of homeless people in London: A complex issue. Substance Use & Misuse. 2003;38(3-6):377-93.

54. MacLellan J, Surey J, Abubakar I, Stagg HR, Mannell J. Using peer advocates to improve access to services among hard-to-reach populations with hepatitis C: a qualitative study of client and provider relationships. Harm Reduction Journal. 2017;14.

55. Cameron A, Lloyd L, Turner W, Macdonald G. Working across boundaries to improve health outcomes: a case study of a housing support and outreach service for homeless people living with HIV. Health & Social Care in the Community. 2009;17(4):388-95.

56. Mc Conalogue D, Maunder N, Areington A, Martin K, Clarke V, Scott S. Homeless people and health: a qualitative enquiry into their practices and perceptions. J Public Health (Oxf). 2021;43(2):287-94.
